# Extra‐pelvic endometriosis: A review

**DOI:** 10.1002/rmb2.12340

**Published:** 2020-07-16

**Authors:** Tetsuya Hirata, Kaori Koga, Yutaka Osuga

**Affiliations:** ^1^ Department of Obstetrics and Gynecology Doai Kinen Hospital Sumida‐ku Japan; ^2^ Faculty of Medicine Department of Obstetrics and Gynecology University of Tokyo Tokyo Japan

**Keywords:** abdominal wall endometriosis, catamenial hemoptysis, catamenial pneumothorax, extra‐pelvic endometriosis, treatment

## Abstract

**Background:**

Extra‐pelvic endometriosis is a rare type of endometriosis, which occurs in a distant site from gynecological organs. The diagnosis of extra‐pelvic endometriosis can be extremely challenging and may result in a delay in diagnosis. The main objective of this review was to characterize abdominal wall endometriosis (AWE) and thoracic endometriosis (TE).

**Methods:**

The authors performed a literature search to provide an overview of AWE and TE, which are the major types of extra‐pelvic endometriosis.

**Main findings:**

Abdominal wall endometriosis includes scar endometriosis secondary to the surgical wound and spontaneous AWE, most of which occur in the umbilicus or groin. Surgical treatment appeared to be effective for AWE. Case reports indicated that the diagnosis and treatment of catamenial pneumothorax or endometriosis‐related pneumothorax (CP/ERP) are challenging, and a combination of surgery and postoperative hormonal therapy is essential. Further, catamenial hemoptysis (CH) can be adequately managed by hormonal treatment, unlike CP/ERP.

**Conclusion:**

Evidence‐based approaches to diagnosis and treatment of extra‐pelvic endometriosis remain immature given the low prevalence and limited quality of research available in the literature. To gain a better understanding of extra‐pelvic endometriosis, it would be advisable to develop a registry involving a multidisciplinary collaboration with gynecologists, general surgeons, and thoracic surgeons.

## Graphical abstract

Abdominal wall endometriosis includes scar endometriosis secondary to the surgical wound and spontaneous AWE, most of which occur in the umbilicus or groin. The diagnosis and treatment of catamenial pneumothorax or endometriosis‐related pneumothorax (CP/ERP) are challenging, and a combination of surgery and postoperative hormonal therapy is essential. Further, catamenial hemoptysis can be adequately managed by hormonal treatment, unlike CP/ERP.

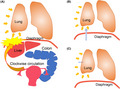

## 1 INTRODUCTION

Endometriosis is defined as a condition in which endometrium‐like tissues are present in organs other than the uterus. It has been reported that 5%‐10% of women of reproductive age have endometriosis.^1^, ^2^, ^3^ It is an estrogen‐dependent inflammatory disease, which causes pelvic pain and infertility. Endometriosis usually involves the ovaries, ligaments, and peritoneal surfaces, and less commonly occurs in the intestine, bladder, abdominal wall, thoracic cavity, and other organs.

The etiology of endometriosis has remained mostly unknown, despite much literature and some predominant theories. The most widely accepted theory for the pathogenesis of endometriosis is retrograde menstruation through the fallopian tubes into the pelvic cavity.^4^ Once endometrial cells adhere to peritoneal surfaces, they can grow and invade onto peritoneal structures, under the influence of the hormonal environment, altered immunity, inflammatory responses, angiogenesis, and other factors.^1^, ^2^, ^5^, ^6^, ^7^ A different hypothesis proposes that metaplasia of the coelomic epithelium may also contribute to the development of the disease.^8^, ^9^ Although pelvic endometriosis is often explained by this implantation theory, extra‐pelvic endometriosis, involving the thoracic cavity and abdominal wall, is difficult to explain with this theory alone.^10^ In addition, because endometriosis occurs in sites that are distant from gynecological organs, the diagnosis of extra‐pelvic endometriosis can be extremely difficult and challenging and may result in a delay in diagnosis and appropriate treatment. In a recent systematic review, most of the reported cases with extra‐pelvic endometriosis (84%) were treated by non‐gynecologic clinicians.^10^

Although the exact frequency of each extra‐pelvic endometriosis is unclear, abdominal wall endometriosis (AWE) and thoracic endometriosis appear to be relatively frequent among cases of extra‐pelvic endometriosis.^10^ This review describes the etiology, clinical presentation, methods of diagnosis, and management of extra‐pelvic endometriosis.

## 2 ABDOMINAL WALL ENDOMETRIOSIS

Abdominal wall endometriosis describes the involvement of ectopic endometrial tissues superficial to the peritoneum of the abdominal wall, including lesions secondary to a surgical incision and spontaneous lesions.^11^ The frequency of AWE has been estimated to be 0.04%‐5.5%.^11^, ^12^, ^13^, ^14^ The typical symptom is a painful mass in the abdominal wall, which may be more symptomatic during menstruation. Most AWE cases are associated with prior surgeries.^11^, ^15^, ^16^ A review describing 445 cases of AWE revealed that 57%, 11%, and 13% of cases were associated with a prior cesarean section, hysterectomy, and other surgery, respectively.^11^ Twenty percent of cases were spontaneous with no surgical history. These cases of spontaneous AWE were preferentially found at the umbilicus or in the groin. In this review, AWE was defined to include scar endometriosis, umbilical endometriosis, and inguinal endometriosis, as previous articles.^11^, ^15^

### 2.1 Scar endometriosis

Scar endometriosis is considered to be the most frequent of the abdominal wall endometrioses and may be defined as an iatrogenic endometriosis.^17^ It predominantly occurs at the cesarean scar, followed by uterine surgery scar, and at the laparoscopic port site.^11^, ^15^, ^18^, ^19^, ^20^ Dissemination of endometrial tissue during a cesarean section or uterine surgery is biologically plausible because of the opportunity to inoculate the abdominal wall with endometrial cells from a hysterotomy. Abdominal mass (96%) and pain (87%) are the most common presenting symptoms.^11^ Local pain at the incision site during menstruation has been reported to be the most common complaint ^11^, ^16^.

Scar endometriosis is often palpable as a subcutaneous mass around the surgical scar on physical examination. If palpable, the physical examination should focus on determining if the mass is attached to the anterior fascia.^11^ More detailed examination is required, especially if the patient does not complain of a palpable mass or pain. Ultrasonography, computed tomography (CT), and magnetic resonance imaging (MRI) are all useful for diagnostic imaging.^21^ Ultrasonography is a simple procedure, which often shows AWE as a low‐echo solid mass. MRI and CT are helpful for clarifying the extent of the lesion, fascia involvement, and the depth of lesion invasion.^21^, ^22^, ^23^ On MRI, the presence of blood components within the abdominal wall mass is suggestive of endometriosis.^21^, ^24^ Fine‐needle aspiration can confirm the preoperative diagnosis of AWE, excluding malignancy.^22^

Surgical resection is recommended for appropriate diagnosis and treatment.^11^, ^15^, ^19^, ^25^. In particular, extensive resection is recommended to avoid postoperative recurrence.^11^, ^15^, ^25^ Some authors have suggested that surgical excision with 1‐cm margins on all sides of endometriotic lesion should be optimal.^26^ Endometriotic lesions have been reported to involve the adipose layer (91.4%‐96.9%), fascia (65.7%‐67.2%), and muscular layer (17.2%‐20.7%).^15^, ^19^ It is recommended to resect the lesion with a resection margin appropriate to the extent of the lesion. Imaging tests such as CT and MRI are useful to determine the extent of the lesion before surgery.^22^

The postoperative recurrence rate of AWE is 4.5%‐11.2%,^11^, ^23^, ^27^ lower than that of ovarian endometriosis. To date, there has been no report demonstrating that hormonal therapy is effective for AWE.^22^ Oral contraceptives (OC), progestin, or gonadotropin‐releasing hormone (GnRH) agonists may be effective in improving symptoms and can be an option for patients who do not want surgery.^16^ However, the symptoms are likely to recur after hormonal therapy is discontinued. Given the low postoperative recurrence rate and the low invasiveness of surgery, surgical treatment can be recommended as the first choice.

Malignant transformation of scar endometriosis was reported to have a very poor prognosis.^28^, ^29^ In a review of 48 cases, the mean age at diagnosis was 46 years, 87.5% were associated with cesarean section, and 12.5% were associated with open uterine surgery.^29^ Regarding the histological type, clear cell carcinoma (66.7%) was the most common, followed by endometrioid carcinoma (14.6%). The average time between initial surgery and diagnosis of endometriotic malignant transformation was 19.3 years, which suggested a slow evolution of the disease. We experienced a case of mixed endometrioid and clear cell carcinoma arising from laparoscopic trocar site endometriosis.^30^ The patient was 49 years old at diagnosis, 180 months after laparoscopic surgery for endometriosis. In a review of 21 cases of trocar site endometriosis, the average age at diagnosis was 32 years, and the interval between initial laparoscopic surgery and diagnosis was 20.6 months on average. Given these observations, age and interval from surgery may be associated with malignant transformation even in laparoscopic trocar site endometriosis. Cases where iatrogenic endometriosis has become malignant are very interesting for understanding the natural history of malignant transformation of endometriosis, assuming that the previous operation was the trigger for the development of endometriosis. Malignant transformation involves multifactorial factors such as genetic, immunological, and environmental factors. It has been reported that some iatrogenic endometrioses have a cancer‐driving mutation,^17^ which may be related to the mechanism of malignant transformation. Also, the malignant transformation and its poor prognosis may further emphasize the need to surgically remove the lesion.

### 2.2 Umbilical endometriosis

Umbilical endometriosis includes secondary umbilical endometriosis, which is thought to develop iatrogenically at the port site after laparoscopic surgery,^31^, ^32^, ^33^, ^34^, ^35^, ^36^ and primary umbilical endometriosis, which has no operation history and occurs spontaneously.^37^, ^38^, ^39^, ^40^, ^41^, ^42^, ^43^ Among umbilical endometriosis, primary umbilical endometriosis is more frequent.^44^, ^45^ In a survey of 96 patients pathologically diagnosed with umbilical endometriosis, 66.7% had no history of prior surgery.^44^ Similarly, in a case series review, 67.6% had no history of surgery.^46^ Unlike scar endometriosis, approximately 70% of patients with umbilical endometriosis did not have a history of prior surgery, suggesting a mechanism other than direct contact implantation. The most likely cause of primary umbilical endometriosis is hypothesized to occur by migrating through blood or lymphatic vessels.^46^ A metaplasia theory has also been proposed. Secondary endometriosis, which occurs after laparoscopic uterine surgery or endometriosis surgery, is thought to be due to the possibility that iatrogenic endometrial cells or endometriosis cells were implanted by direct contact.

Symptoms of swelling, pain, or bleeding at the lesions were present in 86.5%‐90.9%, 80.5%‐81.3%, or 44.8%‐49.2% of cases, respectively.^44^, ^46^ Many patients with umbilical endometriosis are diagnosed by physical examination and imaging modalities.^44^, ^47^, ^48^ While transabdominal ultrasonography can show lesions as low echoic lesions, CT and MRI are more useful to preoperatively determine the size and extent of lesions.^49^

The effectiveness of surgical treatment has been reported, but the outcomes of hormonal therapy have rarely been described. Surgical treatment should involve a wide local resection to avoid recurrence and malignant transformation.^38^, ^44^, ^50^ The recurrence rate after local resection of umbilical endometriosis is approximately 10%,^44^ which is considerably lower than the recurrence rate of ovarian endometriosis. In particular, no postoperative recurrence was reported in cases of radical surgery resected to the peritoneum.^44^, ^50^ When performing a wide resection of umbilical endometriosis, the umbilicus disappears from the abdominal wall and can cause cosmetic problems. In such cases, preoperative consultation with plastic surgeons and umbilical reconstruction should be considered.^44^, ^51^

There have been very few reports on hormone therapy for umbilical endometriosis. Several papers have reported that hormonal treatment is inadequate for treating umbilical endometriosis, while other authors have reported that OC and GnRH agonist were effective in improving symptoms.^43^, ^52^ In our recent report, dienogest, a GnRH agonist, and OC were effective for improving symptoms in 91.7%, 81.8%, and 57.1% of patients, respectively.^44^ GnRH agonists were effective in over 80% of patients, but were unsuitable for long‐term administration due to the hypoestrogenic effect. Discontinuation of hormone therapy is likely to cause recurrence of symptoms. Therefore, long‐term hormonal therapy, including OC and dienogest, may be tolerable alternatives for the treatment of umbilical endometriosis.

Accordingly, surgery may be considered the first choice because of the limited postoperative complication and low postoperative recurrence rate. Hormonal therapy, such as dienogest, may be an option if patients do not desire surgery.

To date, four cases of malignant transformation have been reported.^44,53‐5^. The histological types were clear cell carcinoma in one case, endometrioid carcinoma in one case, and adenocarcinoma in two cases. As a preoperative imaging test, positron emission tomography (PET)‐CT was reported to be useful for the diagnosis of malignancy.^54^, ^55^

### 2.3 Inguinal endometriosis

**FIGURE 1:**
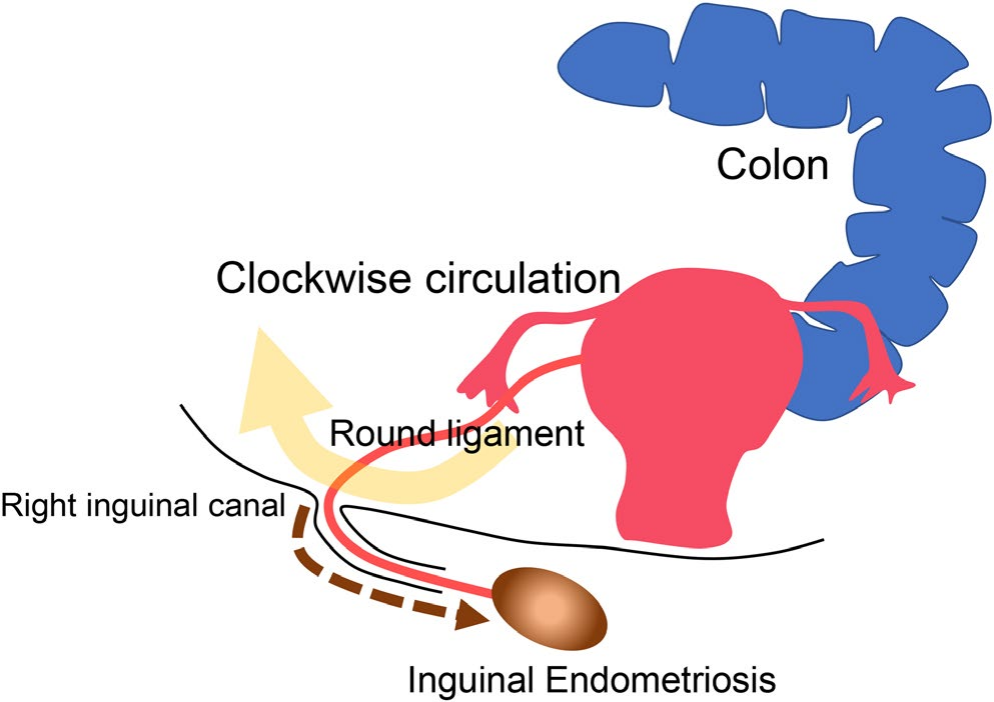
Hypothesized pathogenesis of inguinal endometriosis. The abdominal fluid containing endometrial cells circulates clockwise in the abdominal cavity, and the sigmoid colon blocks the abdominal fluid from entering the left inguinal ring. As a result, the intraperitoneal fluid is more likely to enter the right inguinal ring than the left. Endometriosis also propagates from the pelvis to the groin via the round ligament (brown dot arrow)

**FIGURE 2:**
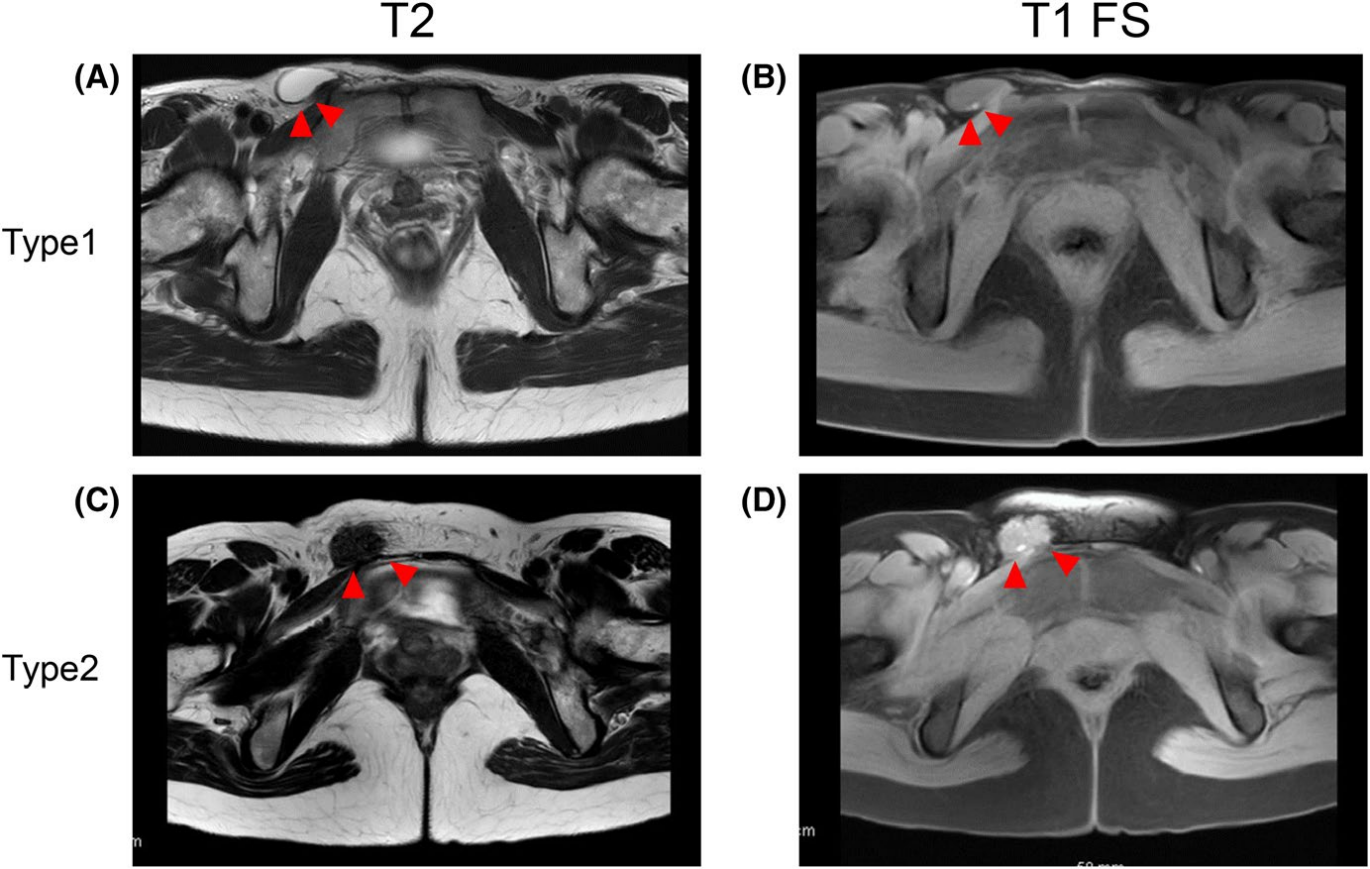
Two types of inguinal endometriosis revealed by magnetic resonance imaging. Red arrowheads denote inguinal endometriosis. A, T2‐weighted axial image shows cystic lesions in the right groin. B, Fat‐saturated T1‐weighted axial image shows the hyperintense nodule in the wall of the cystic lesions. In this case, endometriosis exists and endometriotic lesion exists at the wall of a hernia sac or hydrocele of Nuck’s canal. C, T2‐weighted axial image shows the right inguinal mass (isointense with muscle). D, Fat‐saturated T1‐weighted image shows hyperintensity in the nodule. In this case, endometriotic lesions exist in the solid fibrotic mass

The epidemiology of inguinal endometriosis is poorly understood due to its rarity. The average age at diagnosis is approximately 37 years old.^10^, ^56^, ^57^ Inguinal endometriosis predominantly develops in the right groin,^56^, ^57^, ^58^, ^59^, ^60^, ^61^ and the mechanism is often explained mainly by the theory of reflux of menstrual blood into the pelvis.^62^, ^63^, ^64^ The abdominal fluid containing the endometrium circulates clockwise in the abdominal cavity, and the sigmoid colon blocks the abdominal fluid from entering the left inguinal ring (Figure 1). As a result, the intraperitoneal fluid is more likely to enter the right inguinal ring than the left. It is also speculated that endometriosis propagates from the pelvis to the groin via the round ligament.^62^, ^63^ Inguinal endometriosis occurs in several different forms, including cystic lesions in the hernia sac and Nuck's canal, and solid mass lesions in the extra‐pelvic round ligaments and subcutaneous tissue (Figure 2).^57^, ^65^ MRI is especially useful for preoperative diagnosis of inguinal endometriosis.^57^, ^65^ MRI typically shows two types of inguinal endometriosis on T1‐weighted images, one characterized by hyperintense hemorrhagic cysts and another by a hypointense solid mass with hyperintense hemorrhagic cysts.^65^ In our case series, in 17 of 18 patients who underwent MRI, hyperintensities were seen in T1‐weighted images or fat‐saturated T1‐weighted images.^57^ In particular, fat‐saturated T1‐weighted images were helpful for the diagnosis of inguinal endometriosis. In particular, it is difficult to diagnose endometriosis associated with Nuck's canal hydrocele before surgery, but preoperative diagnosis can be improved by identifying hyperintense sites within the lesion on T1‐weighted and fat‐saturated T1‐weighted images.^57^

Regarding treatment, most reports have described cases with surgical treatment, and reports on cases receiving hormonal therapy alone are very limited. As a radical surgical treatment, an en bloc resection of tumor and the round ligament has been reported.^56^, ^57^, ^62^ The theory is to suppress recurrence by removing the round ligament, which is considered to be the transmission route of endometriosis.^62^ Fedele et al found that endometriotic lesions were located in the extraperitoneal round ligament in all of their cases. These findings may enhance the significance of a radical resection of inguinal endometriosis involving the round ligament. There have been a few reports describing postoperative recurrence rates ranging from 0% to 16.6%.^56^, ^57^, ^62^, ^66^ In these reports, the recurrence sites were similar in all recurrent three cases.^56^, ^57^ A subcutaneous tumor was formed at the site distal to the resection site from the inguinal ring, which suggests that a part of the lesion was newly transplanted during the initial surgery or that a small lesion remained at the distal site of the excised lesion and the round ligament. Given this recurrence mechanism, inguinal endometriosis can recur even after resection of the round ligament, which is presumed to be a translocation pathway. Moreover, it has not been demonstrated whether the combined resection of the round ligament and inguinal endometriosis actually prevents postoperative recurrence. Further study is needed to determine whether round ligament resection is preferable.

Although there have been few reports on the effects of hormone therapy, the administration of dienogest (2 mg/d) was associated with improved groin pain in 6 of 7 patients.^57^ Dienogest can be an option for patients who do not want primary surgery or reoperation after recurrence. Further study is required, because there are limited data on the effects of hormonal therapy on inguinal endometriosis. Accordingly, surgical treatment is recommended as the first choice, because the postoperative recurrence rate is not high and symptoms improve after surgery. In addition, many patients that have been surgically treated by general surgeons for an inguinal hernia or Nuck's canal hydrocele are pathologically diagnosed with inguinal endometriosis after surgery. In such cases, postoperative follow‐up by gynecologist is recommended.^66^

## 3 CATAMENIAL PNEUMOTHORAX AND THORACIC ENDOMETRIOSIS

Thoracic endometriosis (TE) is a major type of extra‐pelvic endometriosis and is characterized by the presence of endometriotic lesions in the thoracic cavity.^67^, ^68^ Manifestation of TE includes catamenial pneumothorax (CP), catamenial hemothorax, catamenial hemoptysis (CH), and lung nodules.^69^ CP is the most common clinical presentation of TE, occurring in 72%‐73% of TE patients, followed by catamenial hemothorax (12%‐14%), CH (7%‐12%), and lung nodule (2%‐6%).^69^, ^70^ CP is defined as recurrent pneumothorax (at least two episodes) associated with the onset of menstruation, within 72 hours.^71^ The most common symptoms associated with CP are chest or shoulder pain, cough, dyspnea, and shortness of breath.^67^, ^72^ Despite the increasing interest and attention to the disease, its etiology remains unclear. Regarding frequency, 20%‐35% of spontaneous pneumothorax in reproductive‐aged women are reported to be CP.^71^, ^73^, ^74^ The onset of CP is usually delayed 5‐7 years after the diagnosis of pelvic endometriosis,^69^ and 50%‐84% of patients with CP have concomitant pelvic endometriosis.^75^ CP has occurred overwhelmingly at the right side, which is over 90% of CP cases.^75^, ^76^ Although several theories have been presented, the etiology and pathogenesis of CP are not fully understood.

### 3.1 Pathophysiology of thoracic endometriosis

**FIGURE 3:**
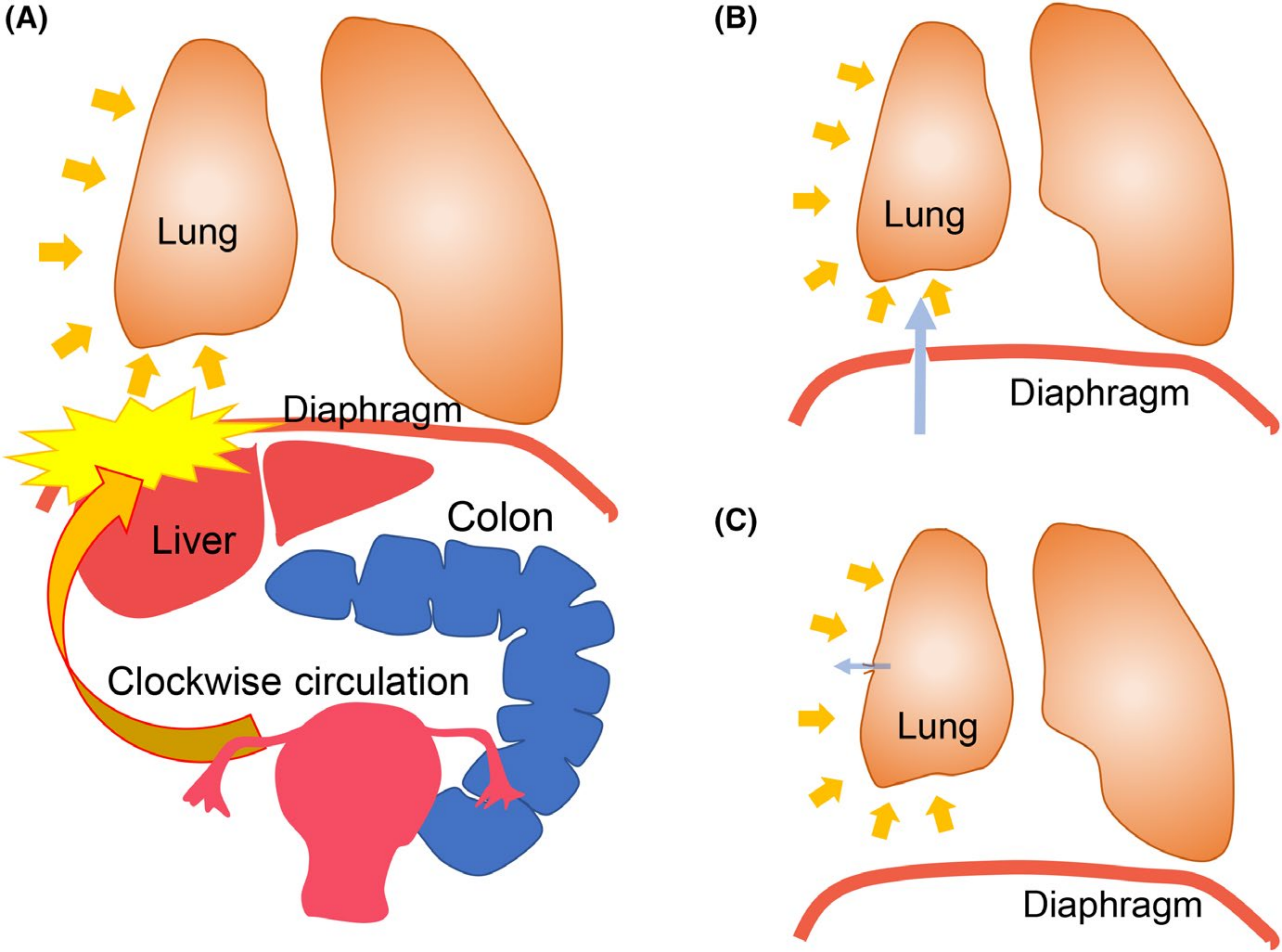
Hypothesized pathogenesis of catamenial pneumothorax (CP) and endometriosis‐related pneumothorax (ERP). A, The clockwise flow of peritoneal fluid containing endometrial cells reaches the right subdiaphragmatic area, while the peritoneal fluid is deviated away from the left hemidiaphragm due to obstruction by the falciform ligament of liver and sigmoid colon. Endometrial cells, which have reached the right hemidiaphragm, adhere to the surface of right hemidiaphragm or migrate into the thoracic cavity through congenital or acquired fenestration in the diaphragm. B and C, Several hypotheses have been proposed regarding how air enters the thoracic cavity. B, Transdiaphragmatic passage of air theory. In this theory, air passes from vagina and uterus into peritoneal cavity through the fallopian tubes. Subsequently, this air enters into thoracic cavity through the diaphragmatic defects, which is congenital or secondary to diaphragmatic endometriosis. C, The visceral pleural and/or superficial parenchymal endometriotic lesion causes the alveoli to rupture and air to flow from the lungs into the thoracic cavity

How endometrial cells reach the thoracic cavity remains unknown. Several theories have been postulated, including coelomic metaplasia, lymphatic or hematogenous spread, or retrograde menstruation with subsequent diaphragmatic migration of endometrial cells. Of these, the retrograde menstruation theory is the most plausible, as it can explain the role of diaphragmatic endometrial implants (Figure 3A).^67^, ^77^ According to this theory, the clockwise flow of peritoneal fluid containing endometrial cells reaches the right subdiaphragmatic area through the right paracolic gutters.^77^ Instead, the peritoneal fluid is deviated away from the left hemidiaphragm due to obstruction by the falciform ligament of liver. It is thought that endometrial cells, which have reached the right hemidiaphragm, adhere to the surface of right hemidiaphragm or migrate into the thoracic cavity through congenital or acquired fenestration in the diaphragm. This theory is supported by right‐sided predominance in CP occurrence. Coelomic metaplasia theory proposed that the endometriotic cells arise by metaplasia of the mesothelial cells of pleura or peritoneal surfaces.^67^, ^73^ This theory can explain plural endometriotic lesions, but cannot explain the right‐sided predominance.^73^ According to the “lymphatic and vascular dissemination theory,” the metastatic spread of endometrial cells through the lymphatic or venous network into the lung or bronchopulmonary area leads to the development of TE. This theory also cannot explain the right‐sided predominance of lesions.^67^, ^78^ Although the above theories have been proposed, none of the theories alone can explain multipresentation of TE, which is most likely multifactorial.

### 3.2 Pathogenesis of catamenial pneumothorax

It is not well understood how endometriosis develops in the thoracic cavity, nor the mechanism by which CP occurs. Several hypotheses have been proposed regarding how air enters the thoracic cavity. The first theory proposes a transdiaphragmatic air passage through the diaphragmatic defects from the uterus and fallopian tubes to the thoracic cavity during menstruation (Figure 3B). This theory is supported by the high concurrent incidence of diaphragmatic defects and right‐sided dominance of diaphragmatic defects. Second, the pleural endometriosis may cause the perforation of alveolar and subsequent air leak (Figure 3C). The third theory is the prostaglandin theory. Prostaglandin F2a, a potent constrictor of the vasculature and bronchioles, increases in plasma during menstruation, which may cause vasoconstriction, bronchospasm, and subsequent alveolar rupture.^79^, ^80^, ^81^

### 3.3 Catamenial pneumothorax and endometriosis‐related pneumothorax (ERP)

Catamenial pneumothorax is defined as recurrent pneumothorax (at least two episodes) associated with the onset of menstruation, within 72 hours.^71^ It is classically known that CP is a typical presentation of TE. Endometriosis‐related pneumothorax (ERP) is defined as pneumothorax due to TE, which is pathologically diagnosed.^80^ Although the majority of CP cases are diagnosed with ERP after surgery, CP can also include primary spontaneous pneumothorax (PSP), which occurs during the perimenstrual period despite non‐ERP. Indeed, it has been found that ERP also occurs at period other than menstruation.^71^ In pathologically diagnosed ERP, 60% occurred during the menstrual period, but 40% occurred during a non‐menstrual period.^82^ Accordingly, CP is not identical to ERP. For these reasons, some authors have recently divided PSP in reproductive‐aged women into four groups: CP and ERP, non‐CP and ERP, CP and non‐ERP, and non‐CP and non‐ERP.^71^, ^74^, ^83^, ^84^ A retrospective classification of 156 women with spontaneous pneumothorax who underwent surgery classified 24 (15.4%) as CP and ERP, 12 (7.7%) as non‐CP and ERP, 13 (8.2%) as CP and non‐ERP, and 107 (68.6%) as non‐CP and non‐ERP.^83^

### 3.4 Catamenial pneumothorax and non‐catamenial pneumothorax

Our group extracted patients with PSP from all national inpatient data and compared the PSP of men and women, and CP and non‐CP. From data of approximately 42 million hospitalization cases, information on 12371 men and 27716 women with PSP was extracted. The age distribution of female patients with PSP had three peaks: 18, around 40, and 80 years, while male patients showed two peaks: 18 and 80 years old. The age of patients with CP had a peak around 40 years, which was identical to the third peak around age 40 in women with PSP. Furthermore, a comparison of CP and non‐CP of reproductive‐aged women revealed that CP occurred approximately 10 times more often on the right side than on the left side, whereas in non‐CP, the left and right frequencies were equivalent. In addition, age, prevalence of endometriosis, number of hospitalizations, and frequency of surgery were significantly higher in CP patients compared to non‐CP patients. Haga et al reported that the following features could distinguish ERP from non‐ERP: right pneumothorax, history of pelvic endometriosis, age of 31 years old or older, and no smoking history.^85^ Accordingly, CP and ERP have quite distinct clinical features from non‐CP and non‐ERP.

### 3.5 Diagnosis of thoracic endometriosis

The diagnosis of TE is fundamentally based on clinical presentation and background. The typical symptoms of TE usually occur and recur during menstruation. The right‐sided predominance of symptoms is also important clue to the diagnosis of CP. However, recognition of these various manifestations of TE can be challenging, particularly when the association between symptoms and menstruations has not been determined.

Although radiographic tests can play an important role in the diagnosis of TE, few reports have been published on the usefulness of these imaging modalities. CT and MRI are the main modalities for detection of TE. CT remains the first‐line imaging method, because it is readily available and inexpensive. CT and chest radiographics are sensitive for the detection of pneumothorax. As CT is poorly specific for TE, its main role is to rule out other diseases. Conversely, MRI has been reported to be superior to CT in diagnosing diaphragmatic, pleural, and hemorrhagic lesions with a reported sensitivity of 83% overall.^86^ MRI findings can also be falsely negative, failing to detect small lesions.^87^ The gold standard for a diagnosis of thoracic endometriosis is video‐assisted thoracoscopic surgery (VATS). Evidence of this disease mostly relies on surgical exploration by VATS, which enables whole intrathoracic exploration including the parietal and visceral pleura, lung, and diaphragm, and subsequently allows to excise or cauterize the detected endometriotic lesions. The investigation of the thoracic cavity has revealed the localization of endometriotic lesions within the thoracic cavity. Nezhat et al reported that TE was found in diaphragm (100%), chest wall (64%), and parenchyma (64%) using the VATS procedure.^72^

The pathological diagnosis of TE can be challenging, because lesions are sometimes so small that glands may not be identified.^88^ Positive histopathological confirmation has been reported to range from 57% to 87.5% ^68^, ^71^, ^73^, ^83^, ^84^, ^89^, ^90^, ^91^. Furthermore, sometimes it is difficult to distinguish endometrial stroma and inflammatory cells by hematoxylin and eosin staining. In such cases, immunohistological examination using markers such as CD10, estrogen receptor, or progesterone receptor is useful.^92^ Recently, our group reported the usefulness of PAX8 as an endometriotic epithelial marker in the diagnosis of extragenital endometriosis, including thoracic endometriosis.^93^ The presence of glands in the resected tissues has been associated with postoperative recurrence.^94^ Therefore, identification of endometriotic epithelia can be clinically important for the postsurgical management of TE. Our group also reported IFITM1 as a novel stromal marker for ovarian and extragenital endometriosis including TE.^95^ The combination of these markers can help identify endometriotic lesions more sensitively.

### 3.6 Treatment of catamenial pneumothorax and endometriosis‐related pneumothorax 

#### 3.6.1 Surgical treatment

When the pneumothorax first occurs, conservative treatment such as needle compression and drainage is usually performed. However, CP is often recurrent and requires further therapeutic management. As described above, VATS may enable the exploration of thoracic cavity and the subsequent surgical procedures such as excision, coagulation, and suturing. Therefore, VATS is a gold standard for diagnosing and treating TE because it allows minimally invasive diagnosis and surgery to be performed simultaneously.

As a surgical procedure, of 370 reported patients with diaphragmatic lesions, partial diaphragm resection and suture were performed in 78.1% (289/370) and coagulation of lesions in 27.2% (101/370) of cases.^10^ Approximately 70% of these procedures were performed using VATS. Deep diaphragm lesions are resected by endoscopic stapling device or by sharp dissection and manual suturing.^72^ However, the use of synthetic mesh has been recommended to close larger diaphragmatic defects.^67^

For cases of pulmonary endometriosis, surgery was performed in 23 of 28 patients and involved pulmonary parenchymal resection (73.9%), lobectomy (21.7%), and pleural resection (4.3%).^10^ Approximately 60% of these operations were performed using VATS.

#### 3.6.2 Pharmacological treatment

Typically, gonadotropin‐releasing hormone (GnRH) agonists are used in first‐line treatments, as they are highly effective in inducing hypogonadotropic hypogonadism and amenorrhea. However, the recurrence rate after hormonal therapy alone was reported to be more than 50%, which is inferior to surgical therapy.^69^ Therefore, surgery should be considered in patients with refractory or recurrent disease under hormonal therapy.^67^ Several studies have suggested the utility of combined surgical and postoperative hormonal therapy to reduce recurrence.^75^, ^76^, ^96^ Alifano et al recommended the combination of surgery and GnRH agonist therapy for 6‐12 months.^71^, ^84^, ^96^ Long‐term use of GnRH agonist induces hypoestrogenic effects leading to menopausal‐like symptoms and osteoporosis, which may result in discontinuation. Discontinuation of hormonal therapy is associated with a high rate of recurrence. Therefore, long‐term hormonal therapy is required for the management of CP or ERP. Recently, our group suggested that dienogest or continuous OC could be an alternative that is appropriate for long‐term use.^76^ These continuous regimens lead to avoidance of cyclic bleeding, which may result in reduction of the frequency of the catamenial symptoms associated with TE.

Recently, an oral GnRH antagonist (elagolix) was reported to be effective for reducing endometriosis‐related symptoms.^97^, ^98^ Unlike GnRH agonists, GnRH antagonists induce rapid and reversible suppression of ovarian hormone levels without the initial 1‐ to 2‐week flare‐up reaction,^98^ which may induce endometriosis‐associated symptoms. In addition, during 12 months of long‐term use, hypoestrogenic adverse effects such as hot flushes may be less than those of GnRH agonists.^99^ Thus, treatment with a GnRH antagonist may be a candidate for effective hormone therapy for TE in the future.

### 3.7 Recurrence of catamenial pneumothorax and endometriosis‐related pneumothorax

The recurrence rate has been reported to be 14.3%‐46.7% during a follow‐up period of 12 months or more after surgery.^68^, ^76^ In a review of 10 studies (478 patients in total), follow‐up for patients with pleural or diaphragmatic endometriosis ranged from 3 to 168 months.^10^ Among these, postoperative pneumothorax recurrence occurred in 29.0% (139/478) of cases of pleural and diaphragmatic endometriosis. However, these results were not stratified by surgery type or postoperative hormone therapy.

Our group reported that the combination of surgery and hormonal therapy tended to have a lower recurrence frequency than either surgery alone or hormonal therapy alone.^76^ For CP and ERP, the combination of surgery and postoperative hormone therapy is currently the most effective management method to reduce the recurrence rate.^67^ However, the recurrence rate after surgery is still high, and there are cases where recurrence of pneumothorax occurs even during hormone therapy after surgery.^76^ Therefore, management of CP and ERP is not satisfactory at this time. Further research is required to develop optimal treatments that will further reduce the recurrence rate.

### 3.8 Differences between catamenial pneumothorax and catamenial hemoptysis

**FIGURE 4:**
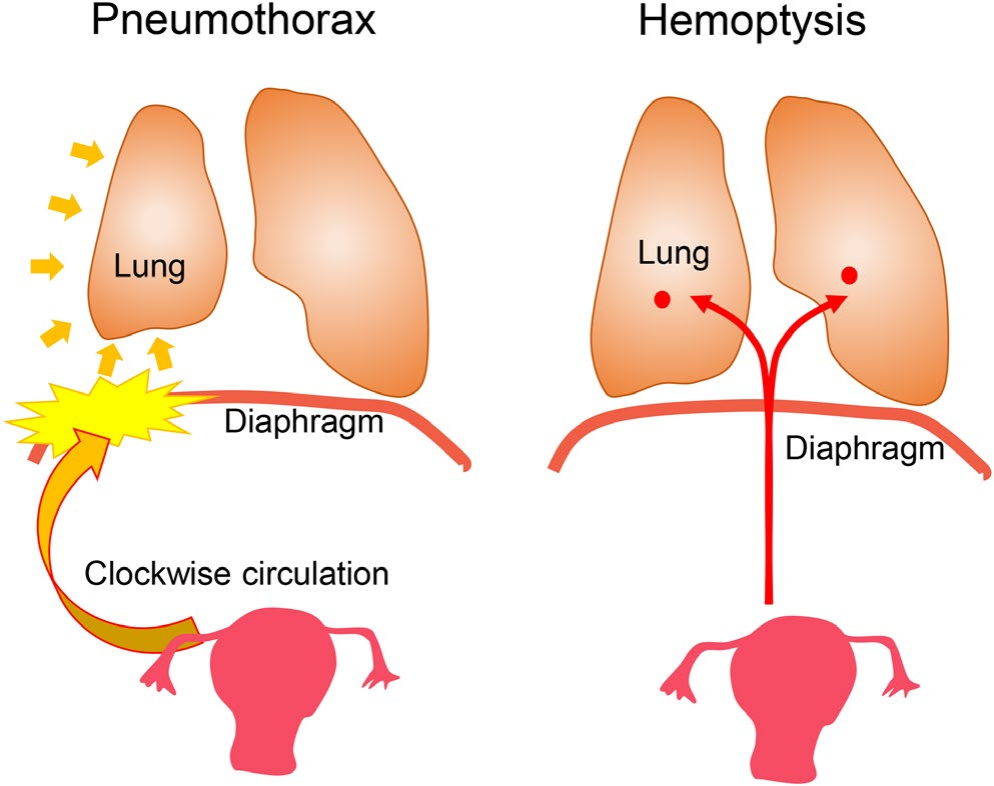
Comparison of hypothesized pathogenesis of catamenial pneumothorax or endometriosis‐related pneumothorax (CP/ERP) and catamenial hemoptysis (CH). A, The clockwise flow of peritoneal fluid containing endometrial cells reaches the right subdiaphragmatic area. Endometrial cells implant on the diaphragmatic surface or enter the thoracic cavity through the defects. B, Intrapulmonary endometriosis, which causes catamenial hemoptysis, is developed by lymphatic or hematogenous microembolization of endometrial cells

Among TE cases, the frequency of CH has been reported to be much lower than that of CP.^69^, ^70^ Although CH is a rare disease, the differences between CH and CP are revealed by a close comparison of clinical features such as patient background and symptoms. According to recent studies, the average age at diagnosis ranged from 25.9 to 29.6 years.^70^, ^76^, ^100^ The age at onset was 8‐10 years younger in CH than in CP/ERP.^70^, ^76^ Secondly, approximately 90% of CP/ERP occurred on the right side, but the occurrence of CH was equivalent in terms of the laterality. Thirdly, CP/ERP had a high probability of concurrent pelvic endometriosis, while CH has a low incidence of pelvic endometriosis,^70^ which suggested CH has a low association with the presence of pelvic endometriosis. These differences may indicate that CH has a different pathogenesis from CP/ERP (Figure 4). Pleural endometriosis, which causes CP/ERP, may be caused by retrograde flow of the endometrial tissue through diaphragmatic defects (Figure 4A). Further, intrapulmonary endometriosis, which causes CH, may develop from the microembolization of endometrial cells. This evidence proposed that the underlying cause of CH was likely to be lymphatic or hematogenous embolization (Figure 4B).^101^

In our recent study, all CH patients experienced symptomatic improvement with hormone therapy, no recurrence during hormone therapy, and consequently no surgical therapy, unlike CP/ERP patients.^76^ Likewise, Kim et al reported complete remission or partial response in all patients treated with hormonal or conservative treatment for CH.^100^ Accordingly, the authors proposed that hormonal or conservative treatment was an adequate first‐line treatment for most patients with CH.^100^ This is in contrast to CP/ERP, which has a high recurrence rate both after surgery and hormonal therapy. Accordingly, CP/ERP and CH are suggested to be distinct entities, although both of them are types of TE.

## 4 CONCLUSION

The number of case reports of extra‐pelvic endometriosis has increased. A review of these reports has partially revealed the features of endometriosis at each site and leads to hypothesize how the causes of endometriosis differ depending on the site (Table 1). Recently, molecular biology techniques using a next‐generation sequencing have also provided clues to the origin of endometriosis and pathogenesis that cannot be elucidated by epidemiological investigations alone.^17^, ^102^, ^103^, ^104^ By continuing basic research and clinical research, the etiology and pathogenesis of extra‐pelvic endometriosis may be further clarified. However, evidence‐based approaches to diagnosis and treatment of extra‐pelvic endometriosis remain enigmatic given their low prevalence and limited quality of research available in the literature. In fact, early diagnosis and treatment of CP/ERP are challenging, and current management still has a high recurrence rate for CP/ERP. At this time, early diagnosis and proper treatment of extra‐pelvic endometriosis require increased awareness of the disease. In addition, patients often go to consultation at a department other than gynecology; thus, it is necessary to optimize standard treatment by conducting a multidisciplinary investigation of accumulated cases. Thus, multidisciplinary collaboration and approaches are important in optimizing patient outcomes. Furthermore, to better understand and treat extra‐pelvic endometriosis, it would be advisable to develop a registry through multidisciplinary collaboration involving gynecologists but also general surgeons, thoracic surgeons, and clinicians managing these patients.

**TABLE 1:** Summary of characteristics of extra‐pelvic endometriosis

	Symptoms	Laterality	Surgical treatment	Postoperative recurrence	Hormonal treatment	The most likely hypothesis on the pathogenesis
Abdominal wall endometriosis						
Scar endometriosis	Swelling, pain, or bleeding at the lesion	N/A	Preferable	4.5%‐11.2%	OC, progestin, or GnRH agonist may be effective by long‐term use.	Endometrial cells are directly implanted via an iatrogenic process.
Umbilical endometriosis	Swelling, pain, or bleeding at the lesion	N/A	Preferable	Approximately 10%	Dienogest, GnRH agonist, or OC may be effective for relieving symptoms.	Spontaneous (endometrial cells migrate to the umbilicus through blood or lymphatic vessels) and iatrogenic (laparoscopic port site)
Inguinal endometriosis	Swelling, pain, or bleeding at the lesion	Predominantly in the right side	Preferable	0%‐16.6%	Dienogest may be effective for relieving symptoms.	Implantation theory (the peritoneal fluid containing endometrial cells enter into the inguinal ring, or endometriosis propagates from the pelvis to the groin via round ligament.)
Thoracic endometriosis						
Catamenial pneumothorax	Dyspnea and chest pain	90% or more in the right side	VATS is a gold standard for diagnosis and treatment	14.3%‐46.7%	Long‐term administration is required.	Endometrial cells reach the right hemidiaphragm and migrate into the thoracic cavity through the defects of diaphragm.
Catamenial hemoptysis	Bloody sputum and chest pain	Equivalent	Mostly not required	Not reported	Effective	Lymphatic and hematogenous embolization of endometrial cells

## DISCLOSURES

*Conflict of interest*: The authors report no conflict of interest. *Human rights statements and informed consent/Animal studies*: This article does not contain any studies with human and animal subjects performed by any of the authors.

## ETHICAL APPROVAL

No ethical approval was needed for this review article.
